# Residual Stress Measurement Using X-ray Diffraction in Friction Stir-Welded Dissimilar Titanium Alloys

**DOI:** 10.3390/ma17071482

**Published:** 2024-03-24

**Authors:** Kapil Gangwar, M. Ramulu

**Affiliations:** 1 Mechanical Engineering, School of Engineering, Wentworth Institute of Technology, Boston, MA 02115, USA; 2 Department of Mechanical Engineering, University of Washington, Seattle, WA 98115, USA

**Keywords:** friction stir welding, dissimilar titanium alloys, residual stress, X-ray diffraction

## Abstract

Surface residual stresses in welded specimens significantly influence properties such as fatigue resistance, fracture toughness, and the superplasticity of joints. In this study, we employed friction stir welding, a well-established joining method, to weld dissimilar titanium alloys. By combining two distinct titanium alloys, we aimed to harness their unique properties when subjected to cyclic loading, impact, or superplastic forming processes. Utilizing X-ray diffraction, macroscopic surface stresses were assessed in dissimilar titanium alloys (Ti-6242 standard grain (SG) and Ti-54M) welded via friction stir welding, assuming a linear lattice distortion. The study accounted for misalignment, significant distortion, and grain refinement in the stir zone. Macroscopic surface residual stresses were quantified on the weld surface and at a depth of 1.5 mm beneath it within a square cross-section (1 × 1 mm^2^) by oscillating the specimen in the (X-Y) direction. The sin^2^φ method, implemented through the LEPTOS^®^ (v7.8) software, was employed for residual stress measurement. The analysis of the results was conducted with respect to different rotation and traverse speeds. It was noted that at the center (CEN) of the weld, commonly referred to as the weld nugget, approximately 50 MPa of tensile stress was observed under the lowest values of both tool rotation speed and traverse speed. Tensile residual stresses were evident at the boundaries and within the stir zone. No discernible pattern was observed at the specified locations. Notably, the resultant values of residual stress, influenced by rotation and traverse speeds, exhibited asymmetry.

## 1. Introduction

Since its establishment in 1991, friction stir welding, along with numerous other variations of solid-state joining, has emerged as a dominant force in the realm of joining technologies across various industries, notably in aerospace [1,2]. Despite having matured and been refined over nearly three decades, the design and structural integrity of friction stir-welded (FSWed) metallic parts require considerations that extend beyond mere alloy selection and processing parameters. These components require thorough metallurgical and mechanical analyses to understand the intricate and hierarchical nature of evolving microstructures. Significant efforts have been dedicated to determining the processing window (rotation and transverse speed) for friction stir welding of similar titanium alloys [3]. A wealth of information, including microstructure analysis and mechanical properties such as tensile strength, fatigue resistance, hardness, and residual stress, is readily available for friction stir-welded similar titanium alloys [4]. However, scarce data exist regarding residual stress values in friction stir-welded dissimilar titanium alloys. In the FSW of dissimilar titanium alloys, migrating elements are anticipated to exert significant influence on crystallographic orientation and lattice strain. Moreover, the severe plastic deformation induced by the tool shoulder (at the weld surface) and pin (within and around the stir zone) must be considered when assessing residual stresses [4].

Residual stresses, whether compressive or tensile, can significantly influence fatigue crack growth. Limited research has been conducted on residual stress measurements and their impact on fatigue crack growth in friction stir-welded titanium alloys [5,6,7,8]. In a study by John et al. on 6 mm thick Ti-6Al-4V sheets subjected to FSW, compressive residual stresses (500 MPa) were found at the root, and tensile residual stresses (approximately 100 MPa) were observed in the center of the weld nugget [7]. Prime et al. have presented contour maps depicting residual stresses on the transverse cross-section of FSWed Ti-6Al-4V sheets [9]. The stress profiles bore a closer resemblance to those observed in fusion welding than the typical two-peak stress distribution reported for FSW in aluminum alloys [10]. Within the stir zone, or nugget, residual tensile stress was evident. A single peak of tensile stresses exceeding 400 MPa was noted just below the surface, corresponding to the tool shoulder. The stress distribution was asymmetric, with higher stresses observed on the advancing side of the weld. The elevated compressive stresses observed at the edge of the plate on the advancing side were likely anomalous and probably resulted from the machining of the original plates [9]. In a research study conducted by Pasta et al., residual stresses in 2 mm thick Ti-6Al-4V sheets were measured using the cut compliance method. The study revealed the presence of high tensile stresses (200 MPa) in the weld nugget (WN), which were counterbalanced by compressive stresses in the adjacent base material [6]. Furthermore, a numerical validation of the residual stress and subsequent fatigue crack propagation (FCP) analysis was performed using AFGROW software v3.0. The results of fatigue tests indicated that the life expectancy for cracks propagating perpendicular to the friction stir-welded (FSW) joint was longer than that for a stress-free base metal specimen [6]. Steuwer et al. investigated the residual stresses in 3-mm Ti-6Al-4V friction stir welds (FSWs) as a function of tool travel speed [11]. They observed that increasing weld traverse speed resulted in a narrower and higher residual stress profile. A 3D thermal-mechanical finite element model for the analysis of FSW of Ti-62A has also been developed with a focus on conventional, stationary shoulder, and bobbin tools [12]. Edwards et al. conducted a study on surface residual stresses using the Hole-Drilling Strain-Gage Method in Ti-6Al-4V sheets welded with thicknesses of 6, 9, and 12 mm [8]. They discovered tensile residual stresses in the longitudinal direction and compressive stresses in the transverse direction. Residual stress profiles were found to be similar for welds of 3 mm, 6 mm, and 9 mm thicknesses, indicating that surface residual stresses developed during welding are independent of thickness. However, the 12 mm weld showed discrepancies with the concurrent results, prompting further investigation to either confirm or challenge existing understanding within the scientific community.

Considering the pursuit of achieving superplasticity in welded panels and addressing parts subjected to varying thermal and mechanical loads, it becomes essential to develop solutions that establish the relationship between fundamental processing parameters (traverse and rotation speed) and the presence of residual stresses in the weld. Furthermore, these solutions must operate within the realms of precision and cost-effectiveness of materials. While mechanical techniques such as hole drilling and methods utilizing nonlinear elastic modulus (such as ultrasonic and magnetic techniques) are effective under certain assumptions regarding the nature of the residual stress field and sample geometry, their spatial depth and resolution capabilities fall orders of magnitude short compared to those of X-ray diffraction.

It is widely acknowledged that compressive residual stresses enhance fatigue resistance under tensile loading conditions, whereas tensile residual stresses offer advantages under compressive loading. However, the literature regarding residual stress measurement in dissimilar titanium alloys is scarce. Therefore, it is crucial to conduct research that can benefit industries, particularly in aerospace, automotive, and biomedical implant applications. By advancing research in the joining of dissimilar titanium alloys, we can facilitate further improvements in these critical sectors.

Over the year, a limited effort has been made to measure the values of the residual stresses in the FSWed similar titanium alloys [8,9,10,11]. However, there is no information available on the residual stress measurement for FSWed dissimilar titanium alloys. Hence, in this article, we have expanded upon the existing knowledge of friction stir welding (FSW) of similar titanium alloys and presented the surface residual stress in FSWed dissimilar titanium alloys on two different surfaces.

Computer-aided design (CAD), coupled with numerical methods and several other experimental methods to measure the residual stresses in metallic alloys, has transformed product development in numerous industries, enabling engineers to validate and analyze complex systems with unprecedented precision and efficiency. This paper explores the XRD $\sin^{2}\varphi$ method to analyze and mitigate residual stresses, demonstrating its efficacy through comprehensive data analysis and experimental validation. Residual stresses, stemming from factors like non-uniform cooling rates and phase transformations during fabrication, are analyzed using $\sin^{2}\varphi$ methods that incorporate key parameters such as material properties (elastic modulus, Poisson’s ratio, and undeformed lattice spacing), manufacturing process details (tool rotation and traverse speed), geometric features, boundary conditions, and environmental factors.

We have emphasized the crystallographic sensitivity of the results, considering the fluorescence associated with titanium alloys and the use of $Cu-K_{\alpha }$ radiation [13]. Furthermore, we discuss the location of diffraction peaks, their intensity and 2θ axis shifts, broadening, and the presence of doublet peaks ($K_{\alpha }$ and $K_{\beta }$), all in the context of Miller indices (hkl) and the influence of sample geometry [14]. We have included details of this novel data analysis approach to elucidate the methodology, particularly in relation to the peaks observed at 102° and 109° corresponding to (203) and (211) reflections, respectively.

## 2. Materials and Methods

The 5 mm thick as-milled sheets of Ti-5Al-4V M (on the retreating (RET) side) and Ti-6Al-2Sn-4Zr-2Mo standard grain (SG) (on the advancing (ADV) side) as provided by The Boeing Company were welded using three rotation speeds (225, 275, and 325 rpm) and traverse speeds (100, 125, and 150 mm/min) to produce a panel measuring $250\times 300\;{mm}^{2}$. Frictional heat-assisted softening, deformation, and eventual solid-state linkage between these two sheets were facilitated by a tapered W-La (tungsten-lanthanum) tool, the dimensions of which are proprietary to The Boeing Company (Seattle, WA, USA). The FSW machine’s automation, placement of the backing anvil, tool insertion, and a 2° tilt on the retreating side, along with controlled argon (Ar) flow, were meticulously managed to ensure defect-free and flashless welds.

While the production of welded panels was graciously facilitated by The Boeing Company, all experiments and analyses were conducted at the University of Washington, Seattle, WA, USA. Specimens were labeled according to the following notation, as shown in Table 1.

**Figure 1:**
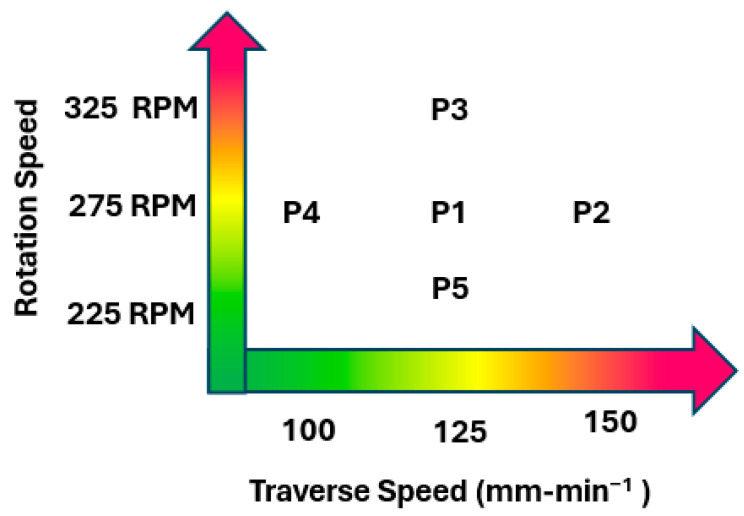
Nomenclature of specimens with respect to rpm and mm/min. For more details about specimens (P1–P5) please refer to Table 1.

Figure 1 illustrates the nomenclature used in the manuscript under the consideration of rotation per minute (rpm) and traverse speed (mm/min).

**Figure 2:**
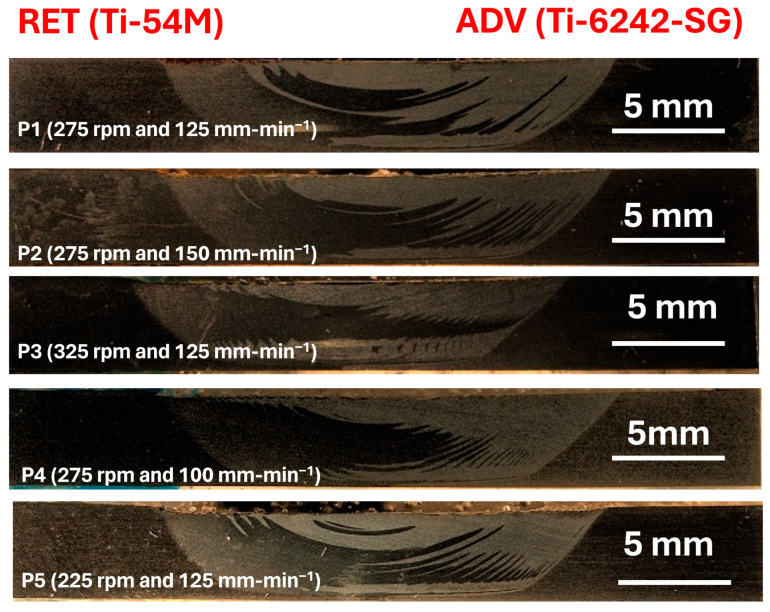
Macrographs of transverse cross-sections of friction stir-welded dissimilar alloys: Ti-54M (RET) and Ti-6242-SG (ADV).

Figure 2 shows the macroscopic images of the welded samples. For relevant information please refer to Table 1. The color represents increasing traverse speed and rotation speed on the x and y axes.

**Figure 3:**
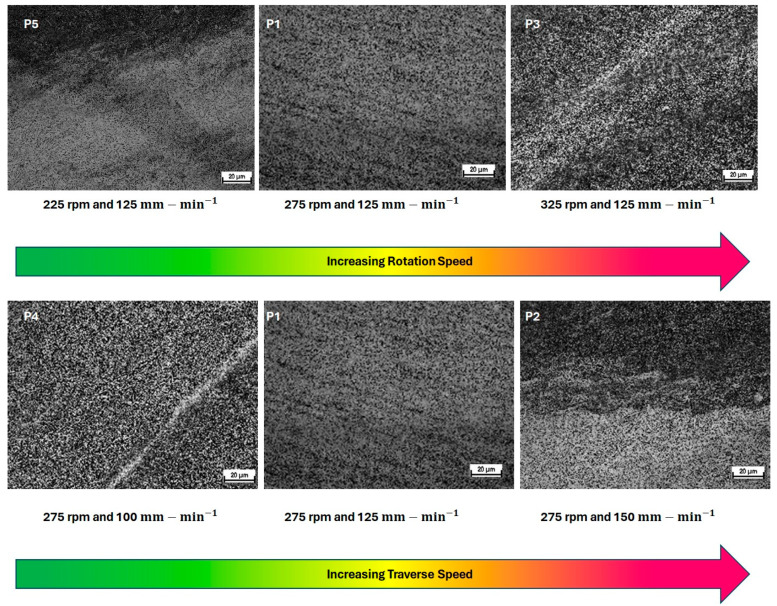
Microstructures in the center of the transverse cross-section of welded samples.

The microstructures in the center of the weld are shown in Figure 3. With Ti-54M being an α−β alloy and Ti-6242 being a near α alloy, the microstructure consisted of a typical combination of equiaxed β grains that decorated grain boundary α, and equiaxed β grains were interspersed with α laths. For a more detailed analysis of the microstructures at multiple locations, readers are recommended to refer to [15].

**Figure 4:**
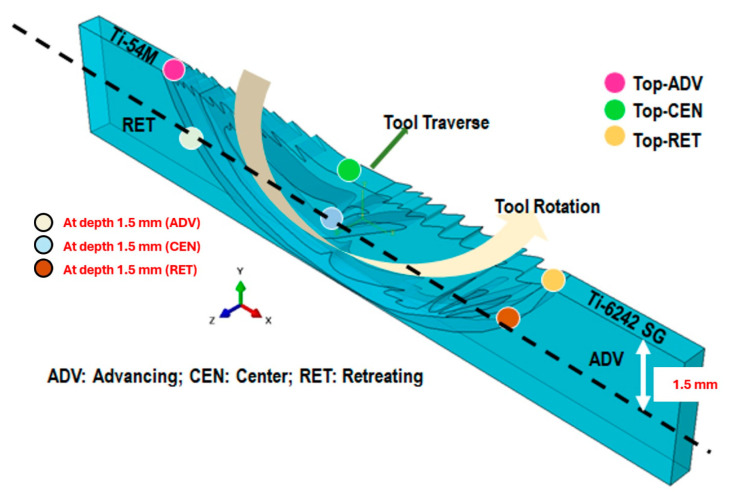
Schematic of friction stir-welded dissimilar titanium alloys and the locations of interest for residual stress measurements. For sample notation, please refer to Table 1.

To determine the impact of rotation and traverse speed in friction stir welding, we conducted experiments on five distinct specimens to assess residual stress. For each specimen, we measured residual stresses both at the top of the weld and across its transverse cross-section, as depicted in Figure 4. Utilizing the X-ray diffraction (XRD) $\sin^{2}\varphi$ method, residual stresses were assessed for two distinct diffraction peaks, (203) and (211). A total of 60 stress tensors were analyzed, culminating in the presentation of Von Mises stress $\left(\sigma_{V}\right)$ values specifically for the (203) peak in this study [16].

## 3. Residual Stress Measurement

Surface residual stresses were assessed on the transverse cross-section of the weld, as depicted in Figure 4. Three key points—(1) at the ADV boundary, (2) at the weld center, and (3) at the RET boundary—were selected for analysis. For the top surface, $\sigma_{yy}$ is zero, and for the transverse surface, $\sigma_{zz}$ is zero.

**Figure 5:**
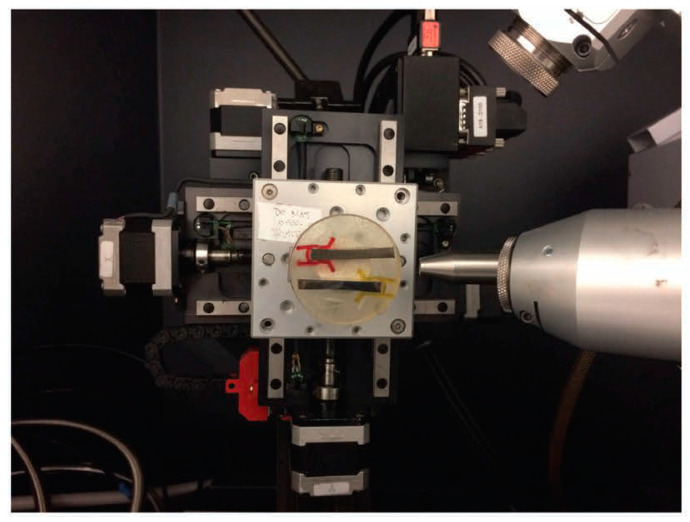
Experimental example of the $\sin^{2}\varphi$ method with an area detector. The instructional label behind the epoxy is white and is irrelevant to the content of this manuscript.

Utilizing diffraction data from area detectors, stress measurement followed conventional methodologies such as the $\sin^{2}\varphi$ method or other algorithms originally developed for conventional diffractometers equipped with point or line detectors. The diffraction profiles resulting from γ-integration were treated similarly to those obtained from a line detector or scanning point detector, as illustrated in Figure 5, which showcases an experimental instance. The residual stress measurement was conducted using a GADDS™ (Bruker, Billerica, MA, USA) microdiffraction system.

**Figure 6:**
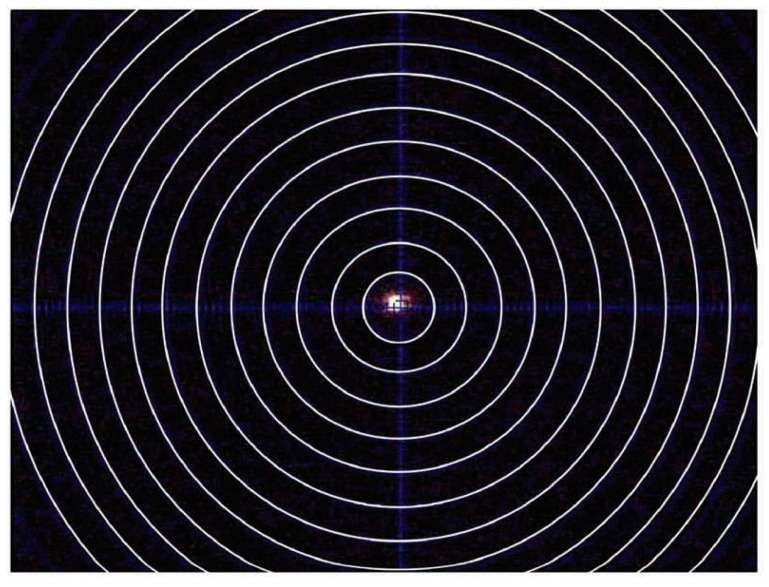
Laser spot with a crosshair. The distance between each circle is 100 μm.

For stress measurement in cases (203) and (211), the diffraction ring of the α phase was utilized. The laser video sample alignment system positioned the inside surface of the spring accurately. Figure 6 demonstrates that the laser beam passing through the spring pitch forms a bright spot on the weld’s surface. Figure 5 and Figure 6 indicate that aligning the sample surface to the instrument center was achieved by overlapping the laser spot with the crosshair. The methodology for stress analysis with XRD involves configuring the diffraction system, planning data collection, correcting data frames, and integrating stress calculations from processed data points. Many concepts and strategies developed for stress analysis with conventional diffractometers are still relevant for XRD.

Stress measurements are primarily conducted on polycrystalline metal parts, typically focusing on high-angle peaks where diffraction intensities may not be very high. Photon counting detectors like multiwire proportional chambers (MWPC) and microgap detectors offer high sensitivity with minimal noise, making them suitable for stress measurement systems using laboratory sources such as sealed X-ray tubes or rotating anode generators. For stress measurements on ferrous metals, Cr or Co radiation is commonly used to prevent fluorescence, and in such cases, multiwire area detectors are preferred. When employing a Mo X-ray source or synchrotron beam, CCD detectors are preferable due to their capability to handle high-energy X-rays and high count rates. Spatial resolution requirements are contingent upon the Full Width at Half Maximum (FWHM) of the diffraction profile. In practice, a FWHM with three to six times the spatial resolution is usually adequate for accurately determining peak positions, provided there are enough counts on the profile. Further reduction in spatial resolution does not necessarily enhance peak position accuracy, as diffraction peak widths for stress measurement are typically broad.

The choice of goniometer and sample stage depends on factors such as sample size, weight, and the stress or stress tensor components to be determined. For instance, a large XYZ stage on a goniometer with two main axes is preferable for handling one normal stress component on a large sample. Co-scanning can be achieved through two main axes in either the θ−2θ or θ−θ configuration. In a vertical θ−θ configuration, co-scanning is achieved by moving the primary beam and detector while keeping the sample stationary, which is advantageous for large samples. The vertical ω−ω configuration requires both the X-ray source and detector to move for the ψ scan. The horizontal θ−2θ configuration offers the advantage of not needing to move the X-ray source, which is particularly convenient for a rotating anode generator. Additionally, moving a heavy 2D detector in horizontal rotation is easier. In 2D stress measurement, the data collection scan can replicate iso-inclination and side-inclination as in conventional methods. The two main axes can provide only the iso-inclination scan (ω scan), while the ψ axis is necessary for the side-inclination scan (ψ). In the conventional method, the ψ scan typically exhibits less variation in the incident angle, resulting in a relatively consistent penetration depth within an interval typically ranging from 5° to 30°. This consistency allows for more uniform and reliable measurements compared to the ω scan. However, despite its advantages, the ψ scan also has limitations. One limitation is that it may not capture detailed information about the microstructure or specific crystallographic orientations within the material. Additionally, the ψ scan may require longer measurement times compared to other techniques, which can impact productivity, especially in high-throughput settings. Moreover, interpretation of the data obtained from the ψ scan may be challenging in complex material systems or when analyzing samples with non-standard geometries. These limitations underscore the importance of selecting the most appropriate scanning technique based on the specific requirements of the analysis. An XYZ stage is crucial for locating the measurement point on the sample and collecting data for stress mapping. A sample alignment device, such as a laser video system, is desired for accurately aligning the measurement spot to the instrument center.

To grasp the sensitivity and identify potential sources of error associated with the measurements, the top of the weld surface was closely examined. The fundamentals of X-ray diffraction and its application in measuring residual stresses are well documented in the literature [17]. Any alteration in the lattice spacing, denoted as d, induces a shift along the 2θ axis. Tensile and compressive residual stresses shift the diffraction peaks to a lower and higher angle, respectively. To assess stress in various directions, the specimen was rotated at three different angles of ψ. For example, when ψ=0 and tensile stress is present in the specimen, it reduces the lattice spacing (thus, slightly increasing 2θ). Conversely, rotating the specimen by known angles ψ increases the lattice spacing beyond the stress-free state, resulting in a decrease in 2θ. Consequently, alterations in the angular position of the diffraction peaks for a given ψ enable the calculation of the stress present on the sample surface lying within the plane of diffraction, which encompasses both the incident and diffracted X-ray beams. Although the incident beam’s diameter was 800 μm, the specimen was oscillated in the X−Y direction, covering a $1\times 1\;{mm}^{2}$ area at the boundaries and within the stir zone. This approach aimed to capture the crystal orientations of as many α laths (in the stir zone), refined equiaxed α grains (on the advancing side), and refined bimodal grains (on the retreating side) as possible, minimizing errors associated with the fluorescence of titanium alloys.

The residual stress measurement system utilized in this study is the Bruker General Area Detector Diffraction System (GADDS), an X-ray diffraction (XRD) system. The system offers two approaches for residual stress measurement: the conventional approach and the two-dimensional (2D) approach. Measurements were conducted with the following configuration: $Cu-K_{\alpha }$ radiation (wavelength of 0.1545049 Å), generator power set at 40 kV/120 mA, and a 0.8 mm collimator with a front pinhole only. For each stress measurement, 24 frames were captured at ω=55°,ψ=0°,20°,40°, and ϕ=0°,45°,90°,132.4°,180°,217°,265°,315°. The data collection time per frame was 120 s, resulting in a total time of 48 min for one stress measurement.

The methodology employed for characterizing the residual stresses observed on the advancing (ADV) and retreating (RET) sides of the weld is outlined in Table 1. Specific stress measurement parameters for a peak observed at $2\theta_{0}=86{}^{\circ}(202)$ are detailed in Table 2.

The calculated frames underwent processing using LEPTOS^®^ (v7.8) software. Particular attention was paid to determining peak location and detecting three monochromatic high-intensity lines: $K_{\alpha 1},\;K_{\alpha 2},\;and\;K_{\beta }$. Given that $K_{\alpha }$ doublets are commonly utilized for residual stress measurement, higher-angle peaks (such as ${\left(203\right)}_{104{}^{\circ}}\;or\;{\left(211\right)}_{110{}^{\circ}}$) were selected to ensure that the data reduction capabilities of Pearson VII distribution functions and a five-point least square parabolic fit fell within the integration limits built into LEPTOS^®^ [18]. For any given peak, such as ${\left(202\right)}_{86{}^{\circ}}$, the following parameters were provided by LEPTOS^®^ and are shown in Table 3 [14].

**Figure 7:**
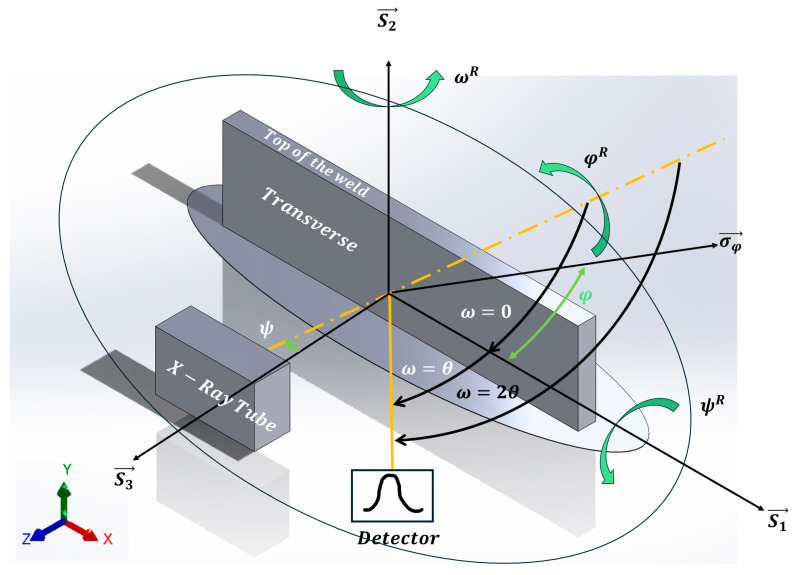
Schematic representation of a specimen goniometer for the $sin^{2}\varphi$ method.

At the top of the weld, the stress component $\sigma_{yy}$ along the XEC direction $\vec{S_{2}}$ is zero. Similarly, for the lateral cross-section of the weld, as depicted in Figure 4 or Figure 7, the stress component $\sigma_{zz}$ along the XEC direction $\vec{S_{3}}$ is zero. Details of the remaining stress tensor values for the ${\left(203\right)}_{102{}^{\circ}}$ peak are provided in the table below.

**Figure 8:**
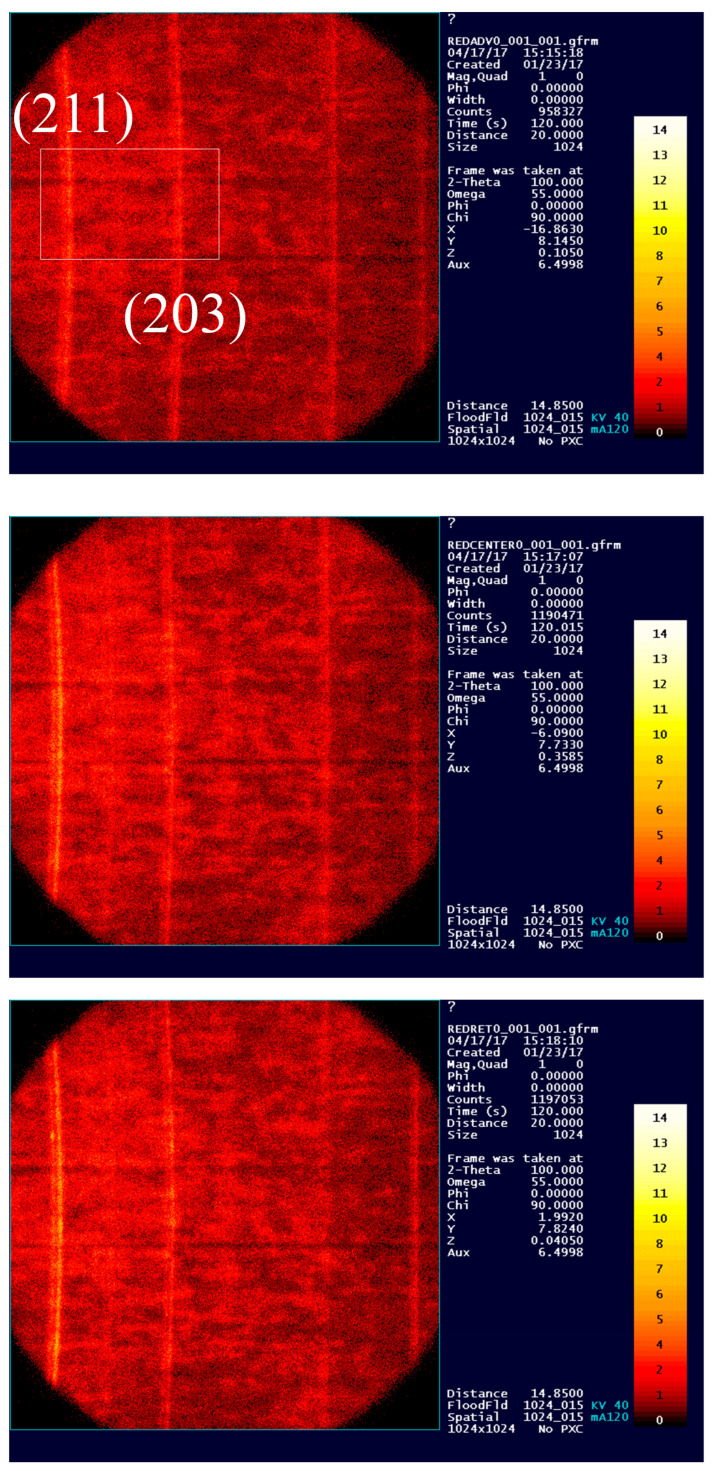
Peak intensity profiles for the General Area Detector Diffraction System (GADDS) with an adapted diffraction scheme for the ADV, CEN, and RET sides. The numbers 0–14 represent the intensity scale in XRD.

Despite our selection of the peak with the least scattering (202), a few sub-regions fell outside the integration range, resulting in significant error values in our calculation [13]. Additionally, the sensitivity of the measurement and the scattering caused by the absorption of the X-ray beam by the detector and the attenuation of the X-ray beam as it penetrates the specimen (expressed by $I(x)=I_{o}e^{-\mu x}$, where $I_{o}$ is the initial intensity and μ is the linear absorption coefficient) affect the lattice spacing (d) for the corresponding peak considered for residual stress measurement. Peaks (203) and (211) constitute the primary constituents of the diffraction pattern in terms of intensity, as depicted in Figure 8. Therefore, residual stress measurements were conducted only for these two peaks. A comparison of stress values ($\sigma_{xx}$ and $\sigma_{yy}$) has been performed for these two peaks across all five specimens (P1–P5).

**Figure 9:**
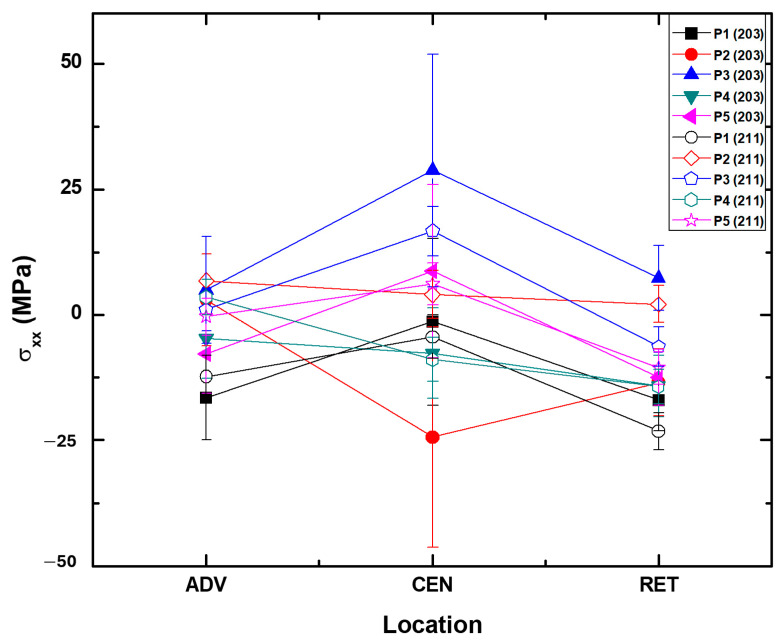
A comparison between residual stresses measured for peaks (203) and (211) for $\sigma_{xx}$ only. A description of P1–P5 can be found in Figure 1 and Table 1. Here, ADV = advancing, CEN = center, and RET = retreating.

To illustrate the variability in the datapoints, we have only included the $\sigma_{xx}\;(or\;\sigma_{11})$ in the following discussion. Readers are encouraged to refer to ref. [15] for $\sigma_{yy}\;(or\;\sigma_{22})$ and further details. Figure 9 presents a comparison of residual stresses for $\sigma_{xx}$ measured at two distinct peaks, illustrating the relational variations within the data. As depicted in Figure 8, a notable disparity in scattering between these two peaks can be observed on the ADV, CEN, and RET sides of the welded sample. To address such occurrences, employing a scandium (Sc) foil as a filter for $K_{\beta }$ peaks separate from corresponding $K_{\alpha }$ peaks can prevent additional integration of the peaks within specified sub-regions and the selected step size 0.01.

While scandium is indeed an expensive metal, its use as a filter offers distinct advantages in X-ray diffraction analysis. One advantage is the high atomic number of scandium, which enables efficient absorption of low-energy X-rays while allowing higher-energy X-rays to pass through. This selective absorption helps in reducing background noise and enhances the signal-to-noise ratio, resulting in improved peak resolution and sensitivity in the diffraction pattern. Additionally, scandium foils are known for their uniform thickness and high purity, ensuring consistent and reliable performance in filtering X-rays. While foils made from basic metals could serve as filters, they may not offer the same level of efficiency and precision as scandium foils, especially in applications where high sensitivity and accuracy are crucial. Another reason is the sensitivity of the results, particularly for peaks observed at higher peaks ${\left(211\right)}_{102{}^{\circ}}\;and\;{\left(211\right)}_{109{}^{\circ}}$.

As illustrated in Figure 9, it is evident that the residual stress values measured by selecting these two peaks [(203) and (211)] are clustered with a significant amount of error for all five specimens (Ti-6242SG and Ti-54M) [14]. However, upon examining the trend in residual stress values, it becomes apparent that, with the exception of specimens P2 and P4, the remaining three specimens follow a consistent profile. Specimens P2 and P4, on the other hand, exhibit an opposite trend in residual stresses compared to the other three. Additionally, an intensity analysis for the two peaks ((203) at 2θ=102° and (211) at 2θ=109°) is presented in Figure 8. It is noteworthy that the peak observed for Ti-6242SG and Ti-54M P1 (275/125) on the retreating (RET) and advancing (ADV) sides exhibits slightly higher intensity values, along with the presence of $K_{\beta }$ with a peak (211). Referring to the sensitivity analysis, it can be inferred that residual stresses measured for this specimen at locations RET and CEN are more reliable with peak (203). Similarly, for location ADV, as depicted in Figure 4, it can be noted that since some amount of $K_{\beta }$ is observed on both peaks, neither of the measurement schemes provides a discrete value for the residual stresses in the absence of an appropriate fluorescence absorption filter (such as scandium foil for titanium and its alloys). Other peaks [(202),(104), and (210)] mentioned in Table 4 do not exhibit sufficient intensity to ensure reliable measurements; hence, all measurements were conducted solely for (203) and (211).

Henceforth, in our calculations, parameters such as step size and peak rejection were meticulously chosen after numerous trials and errors to ensure that integration points on the intensity profiles (as observed in GADDS) align within the integration limits of 2θ. The designated locations chosen for measurements for the five specimens considered in this study are depicted in Figure 4. Results for both the top of the weld and at a depth of 1.5 mm below the top surface on the transverse cross-section are presented individually in this section. A somewhat similar trend was observed in the case of $\sigma_{yy}(or\;\sigma_{22})$ as well as measured from the LEPTOS^®^ (v7.8) software [15].

**Figure 10:**
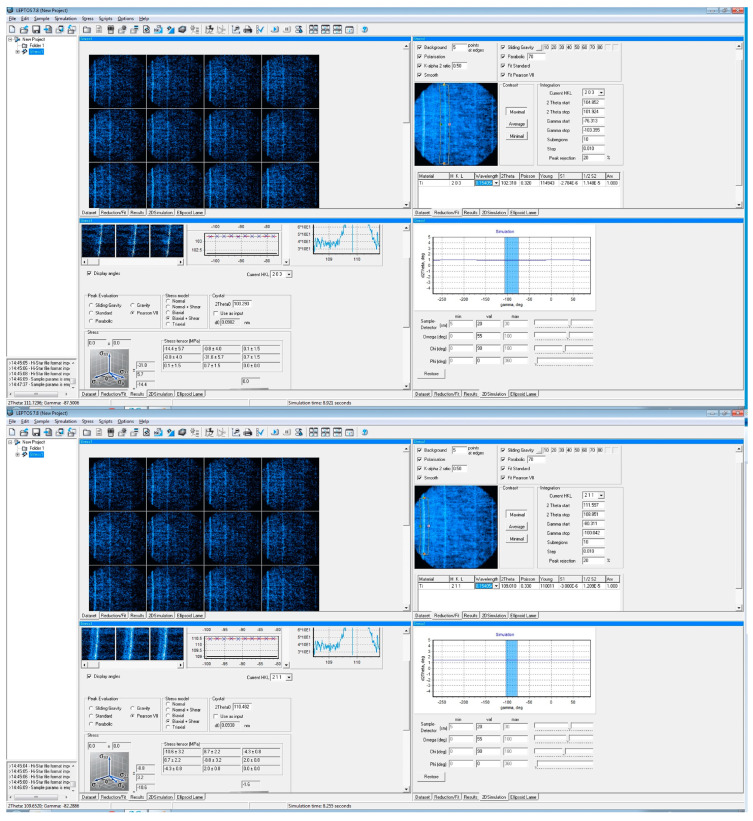
Measurement scheme for residual stresses for peaks (203) and (211).

Considering the variability in the datapoints, for two XRD peaks (203) and (211), obtained at 102° and 109°, respectively, 10 sub-regions with a step size of 0.01 and a peak rejection threshold of 20% were utilized to measure stresses using LEPTOS^®^ (v7.8) software, as shown in Figure 10. Based on the values of stresses in the stress tensor as measured from LEPTOS^®^ (v7.8) software, the values of $\sigma_{V}$ were calculated and are included in the following sections using the LEPTOS^®^ (v7.8) software at the University of Washington, and a methodology is presented in the sections below.

### Sensitivity of Analysis

At high angles, diffraction patterns tend to exhibit a notable amount of scattering in the intensity of reflected beams. Among the five X-ray diffraction peaks observed within the range of (86°–112°; as detailed in Table 4), two peaks, (203) and (211), prominently feature significant volume fractions in friction stir-welded dissimilar titanium alloys [19]. A sensitivity analysis regarding the associated $K_{\beta }$ shift between these two peaks, i.e., (203) and (211), has been conducted and is presented in the following section.

A description of terms used throughout the manuscript is shown in Table 5.

**Figure 11:**
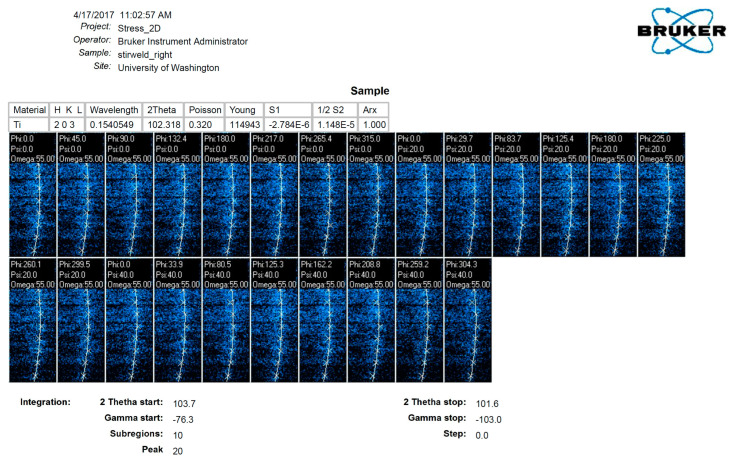
A typical measurement scheme for residual stress for peak (203).

A typical measurement scheme for peak (203) is shown in Figure 11. For more details about the software analysis and the training module, please refer to [20,21].

The sensitivity of the analysis depends significantly on the step size. From these frames, peaks (203) and (211) have been selected based on peak intensity for further analysis. In our discussion, we have focused on the (203) peak due to its significantly higher intensity and minimal presence of the $K_{\beta }$ doublet. This doublet, observed in the X-ray emission spectrum of elements with atomic numbers greater than 30, arises from transitions involving the K shell and indicates variations in X-ray diffraction intensities due to spin-orbit coupling effects in titanium atoms. These effects, stemming from relativistic phenomena in heavy elements like titanium, split X-ray transition energy levels into distinct states. While subtle, the presence of a $K_{\beta }$ doublet in titanium peaks offers valuable insights into the electronic structure and bonding environment within the crystal lattice, aiding material characterization. Moreover, we have elaborated on measurement techniques to underscore how the choice of peak, whether (203) or (211), impacts the sensitivity of the analysis. This emphasis on differentiation within the context of XRD, LEPTOS^®^, and observed values is crucial for a comprehensive understanding and interpretation of our findings. A complete analysis along with 30 stress tensors (five specimens, two peaks, and three locations) for the measurement scheme as shown in Figure 11 can be found here [15].

## 4. Results and Discussion

**Figure 12:**
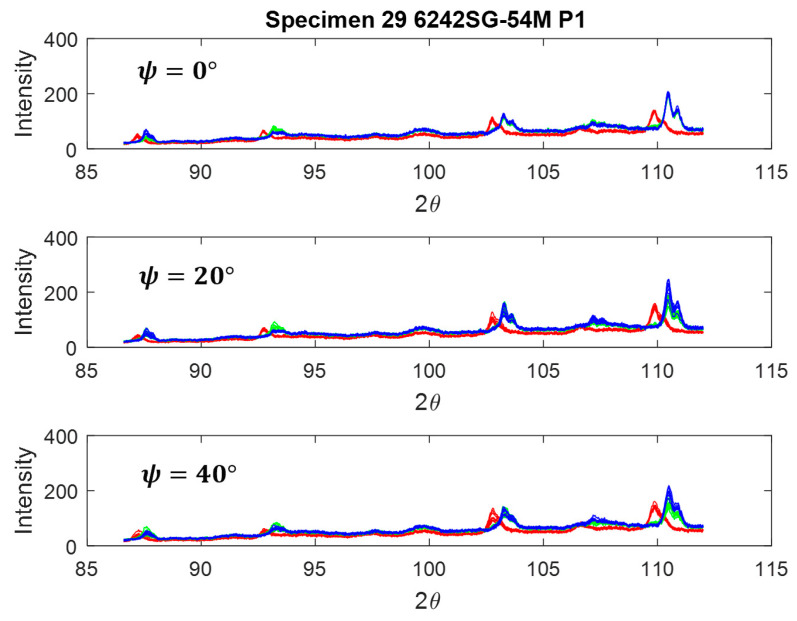
Diffraction patterns for ψ = 0°, 20°, 40° (from top). Red curve corresponds to advancing side, green to center, and blue to retreating side, as measured on the transverse cross-section.

Figure 12 illustrates the intensity profile for three locations on the transverse cross-section of the weld, situated at a depth of 1.5 mm below the top surface of the weld, as designated in Figure 4. The advancing (ADV) side, indicated in red, exhibits shifted and slightly lower intensities of diffraction peaks, suggesting an increase in lattice parameters. Moreover, upon comparison with the texture of this specimen, where ${\left(101\right)}_{\alpha }$ contributes the most to lattice straining in comparison with ${\left(203\right)}_{\approx 103}{}^{\circ}$ and ${\left(211\right)}_{\approx 109}{}^{\circ}$, even a minor change in lattice spacing (and thus 2θ) should signify a peak shift on the ADV side. Visualizing the rotation and traverse in the weld nugget, it becomes apparent that on the retreating (RET) side and in the center of the weld, the material is subjected to greater compression compared to the ADV side. Conversely, on the ADV side, a solid-state linkage exists between the relatively softer (Ti-54M) and harder (Ti-6242 SG) materials. Depending on the processing parameters, especially traverse speed, the stress values and the proportion of Ti-54M present on the ADV side can change significantly.

**Figure 13:**
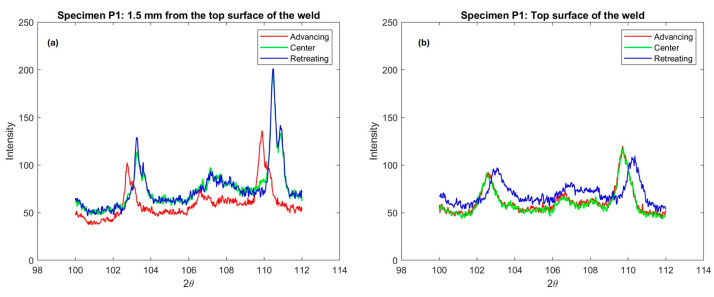
Comparison of two peaks for specimen P1 with the highest intensity on the GADDS intensity profile measured on the (**a**) transverse cross-section of the weld at a depth of 1.5 mm from the top surface of the weld and (**b**) the top surface of the weld.

Based on the shift in the peak intensity profiles observed on the advancing (ADV) side (Figure 12) of the transverse cross-section of the weld, it can be inferred that the ADV side leads to a more uniformly strained lattice compared to the center (CEN) and retreating (RET) sides [22]. To ensure clarity and minimize errors in residual stress measurements, an enlarged view of the inset window (depicted in the ADV frame in Figure 8 (ADV)) is presented in Figure 13 for two different scenarios.

Building upon our earlier discussion regarding the associated $K_{\beta }$ shift in the peaks, we conducted numerous trials by employing various step sizes and integration limits (utilizing Pearson VII distribution functions and a five-point least squares parabolic fit) to achieve convergence. However, for the purpose of discussion, the residual stresses measured for the two peaks, (203) and (211), are presented in the following section.

For specimen P1, an identical comparison of these two peaks was conducted on the top surface of the weld, as depicted in Figure 13b. An intriguing observation in Figure 13b is the positioning of the advancing (ADV) (red), center (CEN) (green), and retreating (RET) (blue) peaks in contrast to Figure 13a. A notably distinct profile is evident on the top surface of the weld. Here, in Figure 13b, we observe a similar pattern between the ADV and CEN sides on the top surface of the weld. The ADV side on the top surface of the weld and the ADV side boundary predominantly consist of Ti-6242 SG, while the RET side remains of the same material (Ti-54M) in both cases. Considering the variation in hardness between the base materials Ti-54M and Ti-6242 SG (with $Ti-54M_{HV}$ > $Ti-6242\;{SG}_{HV}$), and the associated shearing and deformation on the top of the weld ADV side (with Ti-6242 SG positioned atop the weld on the ADV side), it appears to undergo more uniform straining (attributed to the peak shift for ADV and CEN consisting of Ti-6242 SG). Ti-54M on the top surface of the weld experiences relatively uneven straining [23]. Additionally, it is noteworthy that the top surface of the weld undergoes primarily tool rotation, traversing, and shear forces imparted during tool movement. Transformational characteristics seem to play a negligible role in determining the parameters of the strained lattice due to the free surface being exposed to Argon (Ar) gas and temperatures dissipating rather rapidly from the top of the weld. In contrast, on the transverse cross-section, not only deformation but also transformation, dynamic recrystallization, and the appearance of streaks (adiabatic bands) (in shape and size) play a significant role in understanding the variation in residual stress.

Hence, to mitigate measurement errors, peaks (203) and (211) were selected alongside suitable absorption filters, and regression schemes provided in LEPTOS^®^ were utilized. This peak (203) at angle 102° exhibits significantly higher intensity and minimal presence of $K_{\beta }$ doublet, including (202), (104), and (210), along with $\left(211\right)[17]$. Given that these welded specimens are subjected to multiaxial stress states during loading or unloading in real-world applications, it becomes crucial to characterize their stress states in terms of equivalent uniaxial stresses. Thus, Von Mises principal stresses $\left(\sigma_{V}\right)$ were employed to facilitate comparison of XRD results. The ensuing discussion presents the results of the equivalent $\sigma_{V}$ corresponding to the peak (203) [24].

$$Stress\;Tensor\;for\;the\;top\;of\;the\;weld=\left[\begin{array}{ccc} \sigma_{xx} & \tau_{xy} & \tau_{xz}\\ \tau_{xy} & 0 & \tau_{yz}\\ \tau_{xz} & \tau_{yz} & \sigma_{zz} \end{array}\right]$$

$$Stress\;Tensor\;for\;the\;transverse\;cross\;section\;of\;the\;weld=\left[\begin{array}{ccc} \sigma_{xx} & \tau_{xy} & \tau_{xz}\\ \tau_{xy} & \sigma_{yy} & \tau_{yz}\\ \tau_{xz} & \tau_{yz} & 0 \end{array}\right]$$

The exact values of general and principal stresses and corresponding error values for a total of 30 different tensors can be found in ref. [20].

$$\sigma_{V}=\frac{1}{\sqrt{2}}\sqrt{{\left(\sigma_{x}-\sigma_{y}\right)}^{2}+{\left(\sigma_{y}-\sigma_{z}\right)}^{2}+{\left(\sigma_{z}-\sigma_{x}\right)}^{2}+3\left(\tau_{xy}^{2}+\tau_{yz}^{2}+\tau_{zx}^{2}\right)}$$

From principal stresses, the $\sigma_{V}$ can be calculated as follows:

$$\sigma_{V}=\frac{1}{\sqrt{2}}\sqrt{{\left(\sigma_{1}-\sigma_{2}\right)}^{2}+{\left(\sigma_{2}-\sigma_{3}\right)}^{2}+{\left(\sigma_{3}-\sigma_{1}\right)}^{2}}$$

**Figure 14:**
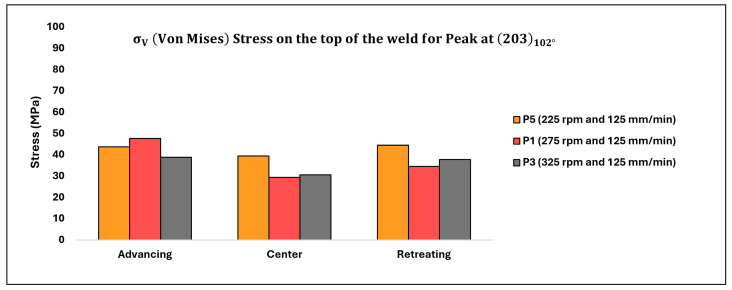
Residual stress distribution on the top surface of the weld for varying rotation speeds.

The corresponding values of $\sigma_{V}$ (in MPa) depicting the impact of rotation speed on the top of the weld are displayed in Table 6 below, with the data also illustrated in Figure 14.

**Figure 15:**
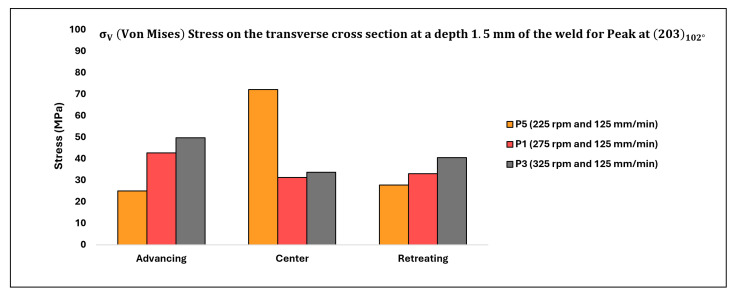
Residual stress distribution on the transverse cross-section of the weld at a depth of 1.5 mm from the top of the weld surface for varying rotation speeds.

Table 7 below displays the corresponding values of $\sigma_{V}$ (in MPa) illustrating the influence of rotation speed on the transverse cross-section of the weld, with the data also depicted in Figure 15.

**Figure 16:**
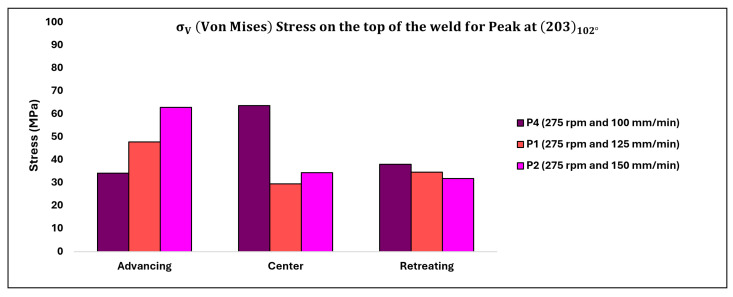
Residual stress distribution on the top surface of the weld for varying transverse speeds.

The corresponding values of $\sigma_{V}$ (in MPa) depicting the impact of traverse speed on the top of the weld are displayed in Table 8 below, with the data also illustrated in Figure 16.

**Figure 17:**
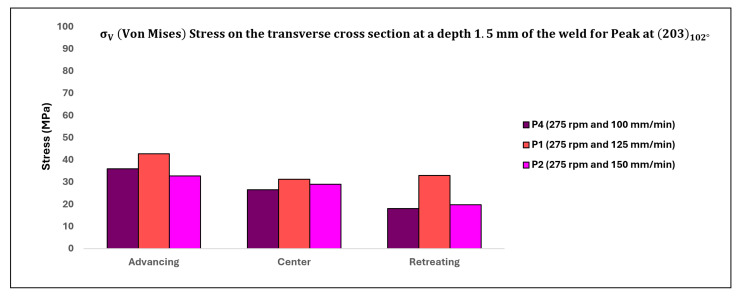
Residual stress distribution on the transverse cross-section of the weld at a depth of 1.5 mm from the top of the weld surface for varying transverse speeds.

Table 9 below displays the corresponding values of $\sigma_{V}$ (in MPa) illustrating the influence of traverse speed on the transverse cross-section of the weld, with the data also depicted in Figure 17.

### 4.1. Effect of Rotation Speed (rpm)

The distribution of residual stresses ($\sigma_{V})$ as influenced by rotation speed on the top surface of the weld and at a depth of 1.5 mm below the top surface of the weld, as observed on the transverse cross-section, is depicted in Figure 14 and Figure 15, respectively, focusing solely on the peak (203) observed at approximately 102° [16]. Analysis of the stress profile on the top of the weld surface (as illustrated in Figure 14) reveals minimal changes in stresses from the advancing (ADV) to center (CEN) to retreating (RET) regions. However, among all three specimens considered, the stresses are relatively higher in the case of the lowest rotation speed. Additionally, stresses on the ADV side are relatively elevated for all three specimens.

At the top surface of the weld, where plunging forces (given that the automation of FSW was displacement-controlled) vary, it is plausible to have higher plunging forces for the lowest rotation speed (P5). Since all measurements were conducted on parts in an equilibrium regime, any asserted vibrations and inhomogeneity in force data were negligible. Consequently, this specimen (P5) likely experienced higher forces on the advancing (ADV) side compared to P1 and P3. As rotation speed increases from 225 (P5) to 275 (P1), stresses in the center (CEN) of the weld decrease significantly compared to the reduction from 275 (P1) to 325 (P3). Notably, β transus temperatures for Ti-6242 SG (ranging between 845 °C and 875 °C; 1553 °F to 1607 °F; located at the top of the weld) were not attained at the lowest rotation speed (225), resulting in higher flow stress in P5’s underlying α phase compared to P1 and P3, where temperatures are closer to the β transus temperature of Ti-6242 SG. On the retreating (RET) side, temperatures are around the β transus temperature for Ti-54M (995 °C to 1005 °C (1823 °F to 1841 °F)) for all three rotation speeds, thus the variation in residual stresses for P1 and P3 is minimal. However, on the RET side, stress is relatively higher for the lowest rotation speed compared to P1 and P3. As the specimen was oscillated 0.5 mm in the x and y directions, the X-ray beam diffracted from both transformed Ti-54M and untransformed Ti-6242 SG present on the RET side at the top of the weld surface.

Stress values at a depth of 1.5 mm from the top surface of the weld in the transverse cross-section exhibit an indeterminate trend. While stress values on the advancing (ADV) and retreating (RET) sides increase with higher rotation speeds, the trend is reversed in the center (CEN) of the weld. In the CEN, the lowest rotation speed yields the highest stress values. Moreover, the variation between P1 and P3 is relatively lower compared to P5, which could be attributed to the different phases present in the weld nugget. For temperatures exceeding the β transus temperature of both alloys, varying thicknesses of α laths are likely to form (depending on the cooling rate), resulting in different residual stresses in the weld nugget [25]. The higher values of stresses in the CEN for the lowest rotation speed suggest the presence of phases with higher flow stress, such as the α phase, predominantly derived from untransformed Ti-6242 SG. On the ADV and RET sides, a similar trend of residual stresses is observed: as rotation speed increases, residual stress values increase. This phenomenon can be explained by the prevailing temperatures: as rotation speed increases, temperatures rise, leading to an accelerated cooling rate. Consequently, the emerging α laths exhibit increasing aspect ratios from P5 to P1 to P3, providing greater resistance to deformation and resulting in higher residual stresses with increasing rotation speed.

### 4.2. Effect of Transverse Speed (mm-min^−1^)

The distribution of residual stresses ($\sigma_{\varphi })$ as affected by traverse speed on the top surface of the weld and at a depth of 1.5 mm below the top surface of the weld, observed in the transverse cross-section, is illustrated in Figure 16 and Figure 17, respectively, focusing solely on the peak (203) observed at approximately 102°. Upon comparing Figure 15, Figure 16 and Figure 17, an intriguing observation emerges: the impact of traverse speed is more pronounced on the top surface of the weld, while the effect of rotation speed is more prominent at a depth of 1.5 mm below the top surface of the weld.

Examining the residual stress variations on the top surface of the weld, as depicted in Figure 16, reveals that as the traverse speed increases, the stresses on the advancing (ADV) side also rise. Since the rotation speed remains constant, it is likely that the advance per revolution (APR, v/ω) is lowest in the case of P4. Consequently, the spacing between two consecutive tool marks is relatively narrower for P4 compared to P1 and P2. Consequently, the evolving residual stresses are more confined for P4 on the ADV side compared to P1 and P2. Additionally, the morphology of tool marks merits consideration: while tool marks are evenly spaced for P2, rather irregular tool marks are observed for P4. Hence, besides the attenuation of the X-ray beam and fluorescence of titanium, geometric errors in the measurements need to be addressed to enhance the quality of the results.

On the retreating (RET) side and in the center (CEN), a somewhat opposite trend is observed compared to the ADV side. On the RET side (where rpm = 275), the effective flow stress provided by the phases is nearly equivalent (since temperatures are uniform), so variation in residual stress values on the RET side is minimal. Conversely, in the CEN of the weld, relatively higher values of residual stresses are noted for P4 compared to P1 and P2. Presently, the cause of this behavior remains uncertain. It is anticipated that the location of the measurement may have been impacted by geometric errors associated with the advance per revolution. To ensure accuracy, the specimen was intentionally marked by the tool marks to capture genuine residual stresses without introducing additional stresses from machining. These geometric irregularities could be attributed to the positioning of the laser spot between tool marks, as depicted in Figure 6 of our manuscript.

Another potential explanation for this phenomenon is that the lower transverse speed causes a greater portion of the plasticized material from the advancing side (ADV) to accumulate in the weld nugget without the formation of adiabatic shear bands, as observed in Figure 2.

At a depth of 1.5 mm from the top surface of the weld, as observed in the transverse cross-section, residual stresses are depicted in Figure 17. An intriguing aspect is that in the case of P1, the stresses are relatively higher at all three locations compared to P4 and P2. On the advancing (ADV) side, as the traverse speed increases, the amount of Ti-54M (in terms of streaks) also increases, resulting in a higher fraction of α laths in the case of P2 (the highest traverse speed). However, due to the oscillatory setup of the goniometer, it can be inferred that the grains diffracting the X-ray beams are not entirely composed of Ti-54M. Hence, despite the highest resistance provided by the α laths, the residual stress values are relatively lower for P2. In the case of P1, where the traverse speed is moderate, there is likely to be more diffraction from Ti-54M streaks than Ti-6242 SG. Conversely, in the case of P4, with the lowest traverse speed, no streaks of Ti-54M are observed on the ADV side. However, since temperatures exceed the β transus temperature on the ADV side for Ti-6242 SG (due to rpm = 275), the aspect ratio of α laths and the presence of equiaxed α grains are likely to be higher compared to P2.

At the center of the weld nugget, the primary constituent of the microstructure is Ti-54M, with some cross-linked Ti-6242 SG present. Depending on the oscillation scheme, only minor variations in residual stresses are observed for all three specimens. On the retreating (RET) side, the highest residual stresses are observed for moderate traverse speeds. Although the shearing forces are not clearly evident at this location, it is expected that there would be a higher exposure time (for evolving temperatures at rpm = 275) in the case of P4. Consequently, the resultant recrystallization of the refined bimodal microstructure on the RET side is highest for P4 compared to P1 and P2. Once again, according to our reasoning, the stresses should have been highest in the case of P2 on the RET side; however, at this point, we are uncertain about the observed values.

## 5. Conclusions

In this study, surface residual stresses on both transverse and longitudinal surfaces of friction stir-welded dissimilar titanium alloys were investigated using X-ray diffraction. The alloys involved were Ti-54M (on the retreating side) and Ti-6242 SG (on the advancing side), welded at three different rotation and traverse speeds. By analyzing the intensity profiles from the General Area Detector Diffraction System (GADDS) and considering associated errors such as fluorescence and $K_{\alpha 2},K_{\beta }$ doublets, two prominent peaks were selected for measuring residual stress values in the higher 2θ regime: (203) at 102° or (211) at 109°.

Key findings and conclusions drawn from our analysis of the residual stresses, particularly for peak (203), include:

1. On the advancing side, where higher temperatures prevail due to the rotational and transverse vectors being in the same direction, more uniform strains were recorded, as indicated by a shift in the peak on the 2θ axis.
2. Peaks (203) and (211) were chosen for residual stress measurement due to their significantly higher intensity compared to other peaks like (202), (104), and (210). To mitigate the effects of fluorescence produced by titanium alloys when using $Cu-K_{\alpha }$, a collimator corresponding to the peak with the least $K_{\beta }$ shift was adopted for measurement, ensuring consistent error levels across all cases. In each case, a peak (203) at approximately 103° was selected to ensure consistency in error considerations and to accurately track the pattern of residual stress variation.
3. Rotation variation notably influenced residual stresses, particularly in the center of the weld (at a depth of 1.5 mm from the top surface on the transverse cross-section). Traverse speed had a more pronounced effect on residual stress variation at the top surface of the weld.
4. Tensile residual stresses were observed at all locations investigated. The presence of evolving phases, the advance per revolution, and the β transus temperature of each alloy had significant effects on the residual stresses observed.

Given the significant fluorescence in the diffraction patterns for titanium with $Cu-K_{\alpha }$ X-ray beams, it is recommended to adjust for $K_{\alpha 2}$ and $K_{\beta }$ doublets and to employ proper filters during analysis.

## Figures and Tables

**Table 1:** Specimen notation followed in this study.

Specimen	Rotation (rpm)	Traverse (mm-min^−1^)
P1	275	125
P2	275	150
P3	325	125
P4	275	100
P5	225	125

**Table 2:** Recipe for residual stress analysis using X-ray diffraction.

Number of Runs	ω (in °)	Ψ (in °)	Angle Coverage (in °)	Number of Frames	Time (s)
1	55	0	45	8	120
2	55	20	45	8	120
3	55	40	45	8	120

**Table 3:** Residual stress parameters for a specific peak (202) observed at $2\theta_{0}\approx 86{}^{\circ}$.

Material	hkl	Wavelength	2θ(in°)	Poisson Ratio (ν)	Young’s Modulus (E) (MPa)	$\vec{S_{1}}$	$0.5*\vec{S_{2}}$
Ti	202	0.1540549 Å	86.73	0.33	111.982	−2.95 × 10 ^−6^	1.19× 10 ^−5^

**Table 4:** Intensity versus 2θ plot for the observed peaks during measurement.

Peak Number	Angle	d(Å)	I%(f)	(h k l)	θ(°)	1/(2d)	2π/d
1	87.576	1.1132	1.8	(2 0 2)	43.788	0.4492	5.6445
2	93.25	1.0597	1.4	(1 0 4)	46.625	0.4718	5.929
3	103.304	0.9822	3.2	(2 0 3)	51.652	0.5091	6.3971
4	107.132	0.9574	1	(2 1 0)	53.566	0.5222	6.5625
5	110.43	0.9379	5.6	(2 1 1)	55.215	0.5331	6.6992

**Table 5:** Description of terms used in the manuscript.

Term	Description
θ	Angle of incidence or diffraction
d	Spacing between crystal lattice planes
I	Intensity of the diffracted X-ray beam
(hkl)	Miller indices of crystallographic planes within a material
φ	Angle of tilt or rotation of the sample around a specific axis relative to the incident X-ray beam
ψ	Represents the angle of rotation around the sample surface normal, also known as the azimuthal angle
ω	Represents the angle of the sample with respect to the incident X-ray beam
E	Elastic modulus
ν	Poisson’s ratio
$\vec{S_{1},}\;\vec{S_{2},}\;\vec{S_{3},}$	Direction of σ in x, y, and z directions
$\varphi^{R}$	Rotation axis for φ
$\psi^{R}$	Rotation axis for ψ
$\omega^{R}$	Rotation axis for ω

**Table 6:** $\sigma_{V}$ (in MPa) on the top of the weld with varying rotation speeds.

Sample/Location	P5	P1	P3
ADV	43.70435	47.73144	38.84315
CEN	39.50076	29.44503	30.57172
RET	44.52628	34.50043	37.82076

**Table 7:** $\sigma_{V}$ (in MPa) on the transverse cross-section of the weld with varying rotation speeds.

Sample/Location	P5	P1	P3
ADV	25.0054	42.74904	49.72112
CEN	72.16779	31.27795	33.73411
RET	27.74869	33.01651	40.45776

**Table 8:** $\sigma_{V}$ (in MPa) on the top of the weld with varying traverse speeds.

Sample/Location	P4	P1	P2
ADV	34.11803	47.73144	62.78208
CEN	63.58168	29.44503	34.3
RET	37.96999	34.50043	31.78506

**Table 9:** $\sigma_{V}$ (in MPa) on the transverse cross-section of the weld with varying traverse speeds.

Sample/Location	P4	P1	P2
ADV	36.02097	42.74904	32.79954
CEN	26.62536	31.27795	29.00827
RET	18.11408	33.01651	19.78181

## Data Availability

The data for this study are proprietary.
